# Efficient hydrogen evolution by ternary molybdenum sulfoselenide particles on self-standing porous nickel diselenide foam

**DOI:** 10.1038/ncomms12765

**Published:** 2016-09-16

**Authors:** Haiqing Zhou, Fang Yu, Yufeng Huang, Jingying Sun, Zhuan Zhu, Robert J. Nielsen, Ran He, Jiming Bao, William A. Goddard III, Shuo Chen, Zhifeng Ren

**Affiliations:** 1 Department of Physics and TcSUH, University of Houston, Houston, Texas 77204, USA; 2 Materials and Process Simulation Center (139-74), California Institute of Technology, Pasadena, California 91125, USA; 3 Department of Electrical and Computer Engineering, University of Houston, Houston, Texas 77204, USA

## Abstract

With the massive consumption of fossil fuels and its detrimental impact on the environment, methods of generating clean power are urgent. Hydrogen is an ideal carrier for renewable energy; however, hydrogen generation is inefficient because of the lack of robust catalysts that are substantially cheaper than platinum. Therefore, robust and durable earth-abundant and cost-effective catalysts are desirable for hydrogen generation from water splitting via hydrogen evolution reaction. Here we report an active and durable earth-abundant transition metal dichalcogenide-based hybrid catalyst that exhibits high hydrogen evolution activity approaching the state-of-the-art platinum catalysts, and superior to those of most transition metal dichalcogenides (molybdenum sulfide, cobalt diselenide and so on). Our material is fabricated by growing ternary molybdenum sulfoselenide particles on self-standing porous nickel diselenide foam. This advance provides a different pathway to design cheap, efficient and sizable hydrogen-evolving electrode by simultaneously tuning the number of catalytic edge sites, porosity, heteroatom doping and electrical conductivity.

## Abstract (web summary)

There is on-going research into efficient noble metal-free materials for electrocatalytic hydrogen evolution. Here, the authors prepare ternary molybdenum sulfoselenide particles supported on three-dimensional porous nickel selenide foam, and demonstrate the high efficiency of the hydrogen evolving composite.

## Graphical abstract

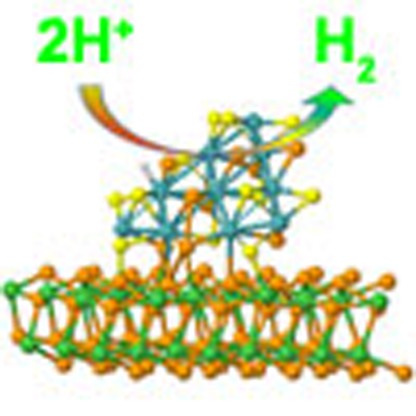

The large consumption of fossil fuels and its impact on the environment make it urgent to develop environmentally friendly and renewable energy sources. Hydrogen (H_2_) is an attractive and promising energy carrier because of its high energy density and no pollution gas emission^1^,^2^. One direct and effective route to generate H_2_ is based on electrocatalytic hydrogen evolution reaction (HER) from water splitting, in which an efficient catalyst is required to ensure the energy efficiency^3^,^4^,^5^. Platinum (Pt)-based noble metals are by far the most active catalysts; however, they are not suitable for large-scale applications because of the high cost and scarcity of Pt on earth^6^. Thus, we aim to identify alternative electrocatalysts based on earth-abundant and cost-effective elements^7^,^8^. Until now, various classes of earth-abundant transition metal compounds are confirmed to be promising candidates^9^,^10^, such as metal sulfides, selenides, phosphides, carbides and the composites. However, thus far most of the catalysts exhibit inferior efficiency to Pt, while many involve complicated preparation methods and multiple steps that increase costs. Great progress has been obtained for HER based on layered transition metal dichalcogenides (LTMDs) such as molybdenum disulfide (MoS_2_) either in the form of crystalline or amorphous states^9^,^10^,^11^,^12^, and even in molecular mimics^13^; however, it remains a challenge to get catalytic performance comparable to that of Pt, which is probably due to the low density and reactivity of active sites, poor electrical transport and inefficient electrical contact to the catalyst^14^,^15^,^16^.

On the basis of the above results, fabricating MoS_2_ or its derivatives into hybrids or composites might be an interesting strategy to promote the catalytic performance^17^. Currently, carbon-based materials are generally used as the catalyst support because of their high surface area and good conductivity^17^,^18^. However, complex catalyst synthesis procedures are typically required. As an alternative, arranging the catalysts into double-gyroid structures with numerous nanopores might lead to improved HER activity because of preferential exposure of catalytic active edges rather than the inactive basal planes^19^. This approach reminds us of the necessity to make three-dimensional (3D) catalysts with high surface area loaded on porous supports, fast proton transfer and greater contact areas with reactants during the catalytic process. Meanwhile, the bottleneck of the double-gyroid structures is the intrinsically poor conductivity of the catalysts. Thus, even though MoS_2_ is established as an effective HER catalyst since 2005 (ref. 11), it is still difficult to obtain satisfactory catalysts in low costs on par with the current Pt catalysts. In the light of these challenges, we conclude that the best strategy is to improve the dispersion and electrical conductivity of these catalysts on the supports and to expose a large number of active edge sites. Furthermore, we consider that arranging two different materials into hybrids might lead to synergistic effects^20^,^21^ that utilize the best properties of each component.

In this work, we propose a strategy of growing ternary molybdenum sulfoselenide MoS_2(1−*x*)_Se_2*x*_ particles with vertically aligned layers on a 3D porous HER-active conductive nickel diselenide (NiSe_2_) scaffold, which takes advantage of the merits of highly conductive support, double-gyroid structures (3D, porous and lots of exposed edge sites) and synergistic effects between two different catalysts. Indeed, we measure excellent HER performance for this hybrid catalyst that is superior to most reported transition metal dichalcogenides (MoS_2_, cobalt diselenide CoSe_2_ and so on).

## Results

### Preparation of 3D porous hybrid electrocatalyst

**Figure 1:**
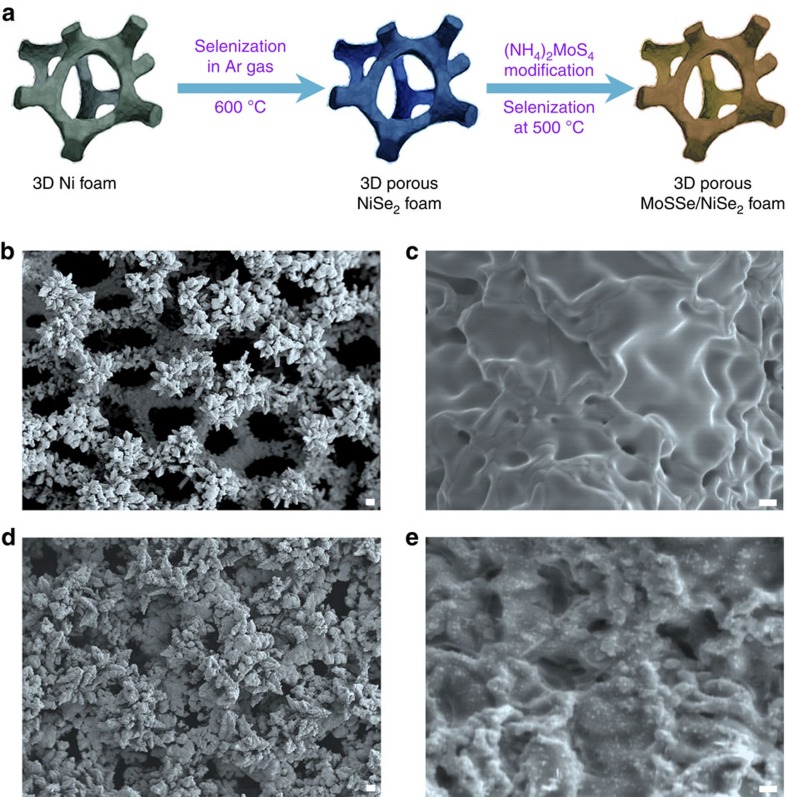
The schematic diagram and morphology characterizations. (**a**) The procedures for growing ternary MoS_2(1−*x*)_Se_2*x*_ particles on porous NiSe_2_ foam. (**b**,**c**) Typical SEM images showing the surface roughness of the NiSe_2_ foam grown at 600 °C from commercial Ni foam. (**d**,**e**) Typical SEM images showing the morphologies of ternary MoS_2(1−*x*)_Se_2*x*_ particles distributed on porous NiSe_2_ foam grown at 500 °C. (**b**,**d**) Scale bar, 50 μm. (**c**,**e**) Scale bar, 1 μm.

To the best of our knowledge, the majority of HER catalysts reported thus far are based on nanostructures (nanoparticles, nanosheets and so on); thus, binder polymers (for example, nafion solution) are necessary to fasten the catalysts on the conducting substrates such as glassy carbon electrodes, which somewhat increases the cost. This problem can be avoided by growing the active catalysts directly on self-standing conducting skeletons as the current collectors^22^,^23^,^24^. The key challenge is to find a suitable 3D supports with high surface area, high porosity and good conductivity. Graphene or carbon nanotube is not feasible because of their high costs. Instead, nickel (Ni) foam is suitable because of its low price, commercial availability and 3D skeleton structure^25^ (Fig. 1a). However, Ni foam is not stable in acidic electrolytes because of corrosion. Interestingly, our previous work shows that direct selenization in Ar atmosphere can convert Ni foam to porous NiSe_2_ foam (Fig. 1b,c, Supplementary Fig. 1 and Supplementary Note 1) that is HER-active and very stable in acid^26^. We find that numerous additional pores are generated in the NiSe_2_, which provides preferential sites for growing LTMD catalysts with high-density active edges^27^. Thus, we propose using 3D porous NiSe_2_ foam as a conductive skeleton to load ternary MoS_2(1−*x*)_Se_2*x*_ catalysts (Supplementary Note 2), thereby utilizing the excellent electrical conductivity, porous structures and high surface area of the NiSe_2_ foam (Fig. 1d,e). Indeed, scanning electron microscopy (SEM; Fig. 1d,e and Supplementary Note 3) images clearly show the uniform distribution of small ternary particles on porous NiSe_2_ foam, which is important for the electrocatalytic performance of LMDT catalysts.

### Structural characterizations of the electrocatalyst

**Figure 2:**
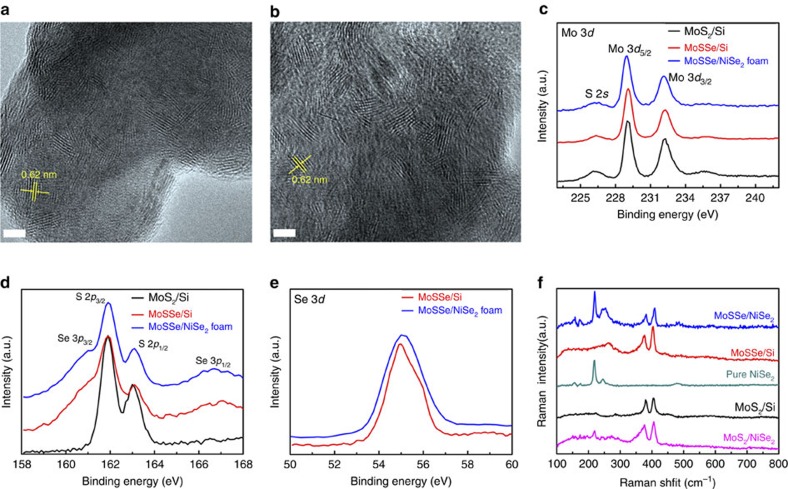
Characterization of the ternary MoS_2(1−*x*)_Se_2*x*_/NiSe_2_ foam hybrid catalysts. (**a**,**b**) TEM images showing the vertical layer orientation of MoS_2(1−*x*)_Se_2*x*_ particles grown on different regions of porous NiSe_2_ foam. Scale bar, 5 nm. (**c**–**e**) Detailed XPS analysis of the Mo 3*d*, S 2*p* and Se 3*d* spectra in different samples, such as binary MoS_2_ particles on Si, MoS_2(1−*x*)_Se_2*x*_ particles on Si and MoS_2(1−*x*)_Se_2*x*_ particles on porous NiSe_2_ foam. (**f**) Raman spectra measured on different samples.

The chemical composition of the as-grown particles was examined using high-resolution transmission electron microscopy (TEM), X-ray photoelectron spectroscopy (XPS), Raman spectroscopy and energy-dispersive X-ray spectroscopy. TEM images (Fig. 2a,b and Supplementary Fig. 2) clearly resolve a large amount of vertically aligned MoS_2(1−*x*)_Se_2*x*_ layers, suggesting that many active edge sites are exposed at the surface of MoS_2(1−*x*)_Se_2*x*_ particles. It is reasonable since the porous structure of NiSe_2_ foam with high surface area is favourable for the growth of layered materials with vertically aligned layers^27^. Meanwhile, XPS spectra in the hybrid reveal the presence of Ni, Mo, S and Se elements (Fig. 2c–e). However, since the Se in NiSe_2_ foam has a similar state to that in MoS_2(1−*x*)_Se_2*x*_, it is difficult to demonstrate the selenization of MoS_2_ on porous NiSe_2_ foam. Instead, to confirm the chemical composition of the molybdenum compound, we put a precursor-decorated Si substrate underlying the NiSe_2_ foam during the second selenization. It is clear that the (NH_4_)_2_MoS_4_ precursor has been converted to a distinctive ternary alloy phase at 500 °C from the prominent Mo, S and Se signals in the XPS spectra^28^ (Fig. 2c–e). Especially in the Raman spectra (Fig. 2f), in comparison with pure MoS_2_ that exhibits two prominent peaks at 380 cm^−1^ (*E*_1g_) and 406 cm^−1^ (*A*_2g_), there is another obvious peak located at 264 cm^−1^ for the samples with a ternary phase, which can be ascribed to the *A*_1g_ mode of the Mo–Se bond^29^. Compared with the Raman mode of the bulk MoSe_2_ crystals (∼242 cm^−1^), the blueshifts of this peak to 264 cm^−1^ suggest a ternary MoS_2(1−*x*)_Se_2*x*_ compound rather than a mixture of two solid phases. This Raman feature is also observed from the ternary phase grown on porous NiSe_2_ foam, which is consistent with previously reported results on ternary MoS_2(1−*x*)_Se_2*x*_ single crystals^29^. By comparing the relative peak intensity between 264 and 380 cm^−1^, we estimate that the atomic ratio between S and Se is ∼1, which is further supported by the energy-dispersive X-ray spectroscopy analysis (Supplementary Fig. 3).

### Hydrogen evolution catalysis

**Figure 3:**
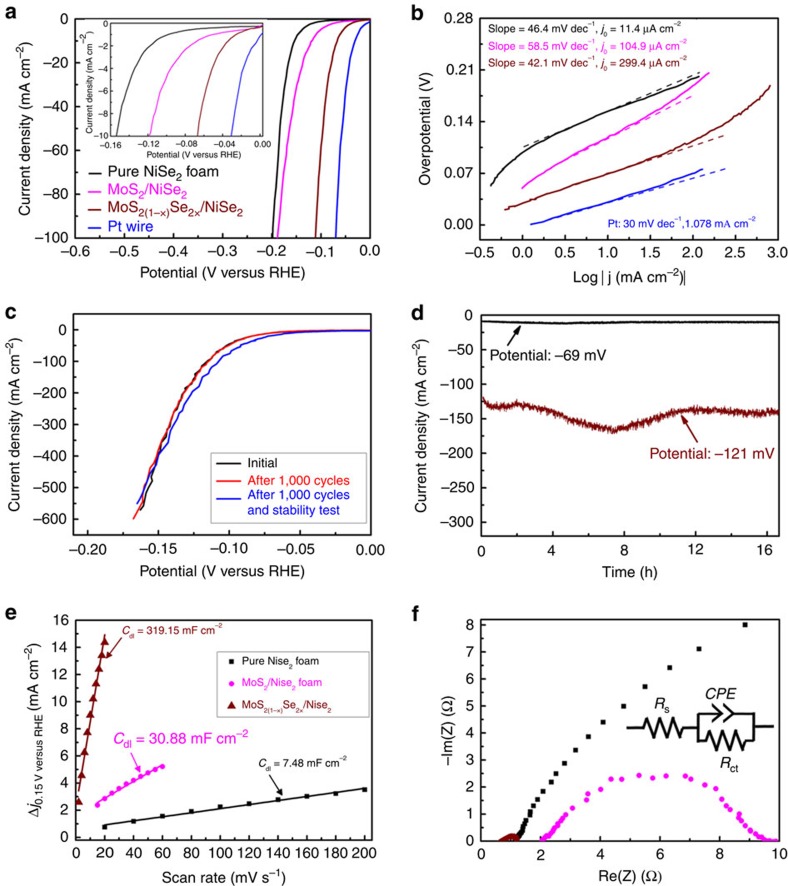
Electrocatalytic performance of different catalysts. (**a**) The polarization curves recorded on MoS_2(1-x)_Se_2x_/NiSe_2_ foam hybrid, MoS_2_/NiSe_2_ foam hybrid and pure NiSe_2_ foam electrodes compared with a Pt wire. (**b**) Tafel plots recorded on the catalysts in **a**. (**c**) Polarization curves showing negligible current density loss of ternary MoS_2(1−*x*)_Se_2*x*_/NiSe_2_ hybrid electrodes initially, after 1,000 CV cycles and after the stability test. (**d**) Time dependence of current densities −10 and −140 mA cm^−2^ recorded on the MoS_2(1−*x*)_Se_2*x*_/NiSe_2_ hybrid electrode under given potentials of −69 and −121 mV, respectively. (**e**) Plot showing the extraction of the *C*_dl_ from different electrodes. (**f**) Electrochemical impedance spectroscopy (EIS) Nyquist plots of different electrocatalysts. The data were fit to the simplified Randles equivalent circuit shown in the inset. The loading of MoS_2(1−*x*)_Se_2*x*_ catalyst is 4.5 mg cm^−2^.

Considering the metallic and porous feature in the NiSe_2_ foam, and the good dispersion and preferential layer orientation of ternary MoS_2(1−*x*)_Se_2*x*_ particles, it is expected that this 3D architecture should have outstanding HER activity, such as low overpotentials, low Tafel slopes and large exchange current densities. To evaluate the catalytic performance of these ternary MoS_2(1−*x*)_Se_2*x*_ particles on 3D porous NiSe_2_ foam, we performed detailed electrocatalytic measurements via a standard three-electrode set-up in a 0.5 M H_2_SO_4_ electrolyte de-aerated with high-purity N_2_. The loading of MoS_2(1−*x*)_Se_2*x*_ catalysts is ∼4.5 mg cm^−2^. Figure 3a shows that the self-standing porous hybrid catalyst can afford a geometric current density of −10 mA cm^−2^ at a very low overpotential of −69 mV for the ternary MoS_2(1−*x*)_Se_2*x*_/NiSe_2_ hybrid electrode (Supplementary Table 1). In contrast, for binary MoS_2_ on NiSe_2_ foam and pure NiSe_2_ foam, overpotentials of −118 and −153 mV are needed to achieve −10 mA cm^−2^, respectively. The catalytic overpotential (−69 mV) of the MoS_2(1−*x*)_Se_2*x*_/NiSe_2_ hybrid is also much lower than those of the best catalysts thus far based on LTMDs MoS_2_ (−110 mV)^18^, WS_2_ (−142 mV)^15^ and WS_2(1−*x*)_Se_2*x*_ (−170 mV)^30^, and first-row transition metal dichalcogenides CoSe_2_ (−139 mV)^24^, NiSe_2_ (−136 mV)^26^ and CoS_2_ (−142 mV)^31^, suggesting that our ternary MoS_2(1−*x*)_Se_2*x*_ particles/NiSe_2_ foam hybrid is an outstanding HER catalyst. Meanwhile, a Tafel slope, which is an inherent property of the catalyst, can be obtained by extracting the slopes from the linear regions in Tafel plots (Fig. 3b). We find that the ternary electrode possesses a smaller Tafel slope of 42.1 mV per decade than that of binary MoS_2_ on NiSe_2_ foam (58.5 mV per decade) and pure NiSe_2_ foam (46.4 mV per decade). In addition, our hybrid catalyst leads to a Tafel slope much lower than many previously reported cheap and efficient HER catalysts in the same electrolyte (Supplementary Table 2). More interestingly, based on the intercept of the linear region of the Tafel plots, the exchange current densities (*j*_0_,_geometrical_) at the thermodynamic redox potential (*η*=0) can be calculated to be 299.4 μA cm^−2^ for the ternary-phase hybrid catalysts. This exchange current density is one to two orders of magnitude larger than those of well-known LTMDs MoS_2_ and WS_2_, or first-row transition metal dichalcogenides CoSe_2_ and CoS_2_ catalysts (Supplementary Table 2). Thus, considering the small overpotential (−69 mV to reach 10 mA cm^−2^), low Tafel slope (∼42.1 mV per decade) and large exchange current density (∼299.4 μA cm^−2^), it is worth pointing out that the catalytic performance of our as-prepared catalyst is superior to most of the MoS_2_-based catalysts.

Aside from a stringent requirement for high HER activity, stability is another important criterion in evaluating the performance of an electrocatalyst. In our experiment, a long-term cyclic voltammetry (CV) test between −0.20 and 0.07 V versus RHE shows no significant degradation of cathodic current densities for the hybrid catalyst after 1,000 cycles (Fig. 3c). Particularly, the cathodic current density for the hybrid catalyst remains stable and exhibits no obvious degradation for electrolysis at a given potential (−69 or −121 mV) for over a long period (>16 h; Fig. 3d), suggesting the potential use of this catalyst over a long time in an electrochemical process. Even after long-term stability and cyclability tests, the catalytic performance of this hybrid catalyst still shows no degradation compared with its initial state (Fig. 3c). In addition, the Faradaic efficiency for hydrogen evolution of this hybrid catalyst was evaluated (Supplementary Note 4). The efficiency is determined to be nearly 100% during 60 min of electrolysis (Supplementary Fig. 4).

To elucidate the origin of the differences in the overall catalytic performance among different catalysts, a simple CV method^15^,^20^,^24^ was utilized to measure the corresponding electrochemical double-layer capacitances (*C*_dl_) for evaluation of the electrochemically effective surface areas (Supplementary Fig. 5). Taking consideration of the direct proportion between the effective surface area and double-layer capacitance, we just need to compare the capacitance values *C*_dl_. By plotting the positive and negative current density differences (*Δj*=*j*_a_−*j*_c_) at a given potential (0.15 V versus RHE) against the CV scan rates, we can directly get the *C*_dl_, which is equal to half the value of the linear slopes of the fitted lines in the plots. As shown in Fig. 3e, the MoS_2(1−*x*)_Se_2*x*_/NiSe_2_ hybrid electrode exhibits a *C*_dl_ value of 319.15 mF cm^−2^, which is one order of magnitude larger than that of the pure MoS_2_/NiSe_2_ foam (30.88 mF cm^−2^), and ∼43 times larger than that of pure NiSe_2_ foam (7.48 mF cm^−2^), demonstrating the proliferation of active sites in the porous hybrid catalyst, which accordingly results in the improved catalytic performance. From these capacitance values, we can roughly calculate the electrochemically effective surface area, and thus the turnover frequency per site (0.030 s^−1^ at 100 mV and 0.219 s^−1^ at 150 mV, see Supplementary Table 3) by using a similar calculation method developed by Jaramillo *et al.*^32^ (Supplementary Note 5). The turnover frequency values are larger than many values reported on MoS_2_-like catalysts, and close to that of transition metal phosphide-based electrocatalysts (Supplementary Table 3). On the other hand, electrochemical impedance spectroscopy was carried out to examine the electrode kinetics under the catalytic HER-operating conditions (Fig. 3f). According to the Nyquist plots and data fitting to a simplified Randles circuit, our results clearly reveal that the charge-transfer resistance (*R*_ct_∼0.5 Ω) for the MoS_2(1−*x*)_Se_2*x*_/NiSe_2_ hybrid is much smaller than that for pure MoS_2_/NiSe_2_ (*R*_ct_∼8 Ω) or for porous NiSe_2_ foam alone (*R*_ct_∼22 Ω). In addition, all the catalysts have very small series resistances (*R*_s_∼0.6−1.2 Ω), suggesting high-quality electrical integration of the catalyst with the electrode.

### Quantum mechanics calculations

**Figure 4:**
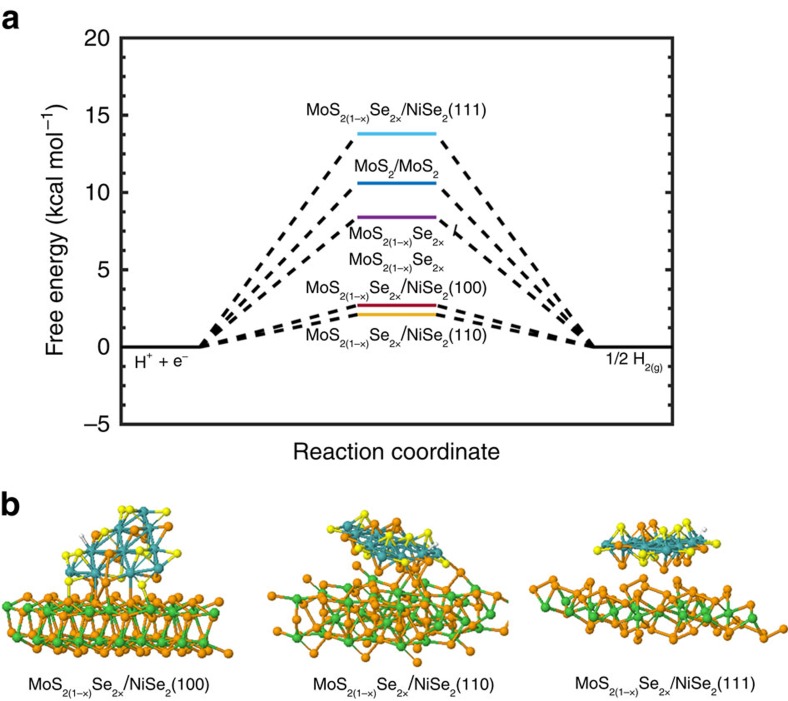
Density functional theory calculations. (**a**) Calculated adsorption free energy diagram for hydrogen (H*) adsorption at the equilibrium potential for MoS_2(1−*x*)_Se_2*x*_/NiSe_2_ hybrid, binary MoS_2_ and ternary MoS_2(1−*x*)_Se_2*x*_ catalysts. (**b**) Intermediate structures of hydrogen-bound MoS_2(1−*x*)_Se_2*x*_ /NiSe_2_ (100), MoS_2(1−*x*)_Se_2*x*_/NiSe_2_ (110) and MoS_2(1−*x*)_Se_2*x*_ /NiSe_2_ (111).

To understand the improvement on the catalytic hydrogen evolution of the MoS_2(1−*x*)_Se_2*x*_/NiSe_2_ hybrid catalyst, quantum mechanics calculations at the density functional theory (DFT) level (PBE-D3 flavor, see Supplementary Note 6) were performed to calculate the binding free energies of hydrogen on the Mo atom^11^,^23^. Although it was originally suggested that the edge S atom is the catalytic atom in hydrogen evolution on MoS_2_ (ref. 11), we find that H_2_ formation going through the Mo atom via the Heyrovsky reaction^33^ has a lower barrier than the Heyrovsky and Volmer^34^ reaction on the S atom. Therefore, we use a lower hydrogen-binding energy on the Mo atom as the indicator of a lower barrier in the Heyrovsky step. Since there are various exposed facets in our as-prepared NiSe_2_ foam (Supplementary Fig. 1), we modelled the reaction on the simple low-index (100), (110) and (111) surfaces of NiSe_2_. Molybdenum dichalcogenide with Se:S ratios of 0:1, 1:1, 1:0 are modelled, and, in the 1:1 case, the S and Se alternate above and below the plane to avoid strain. As shown in Fig. 4a, Δ*G*_H_* is 8.4 kcal mol^−1^ for hydrogen adsorbed on MoS_2(1−*x*)_Se_2*x*_/MoS_2(1−*x*)_Se_2*x*_, which is more reactive than MoS_2_/MoS_2_ with a *ΔG*_H_* of 10.6 kcal mol^−1^, agreeing with the reported experimental results (Supplementary Figs 6 and 7)^35^,^36^. In contrast, once the MoS_2(1−*x*)_Se_2*x*_ particles are hybridized with porous NiSe_2_ foam, the relevant *ΔG*_H_* on MoS_2(1−*x*)_Se_2*x*_/NiSe_2_ (100) and MoS_2(1−*x*)_Se_2*x*_/NiSe_2_ (110) are further decreased to 2.7 and 2.1 kcal mol^−1^, making these hybrid catalysts much more active than MoS_2_/MoS_2_ and MoS_2(1−*x*)_Se_2*x*_/MoS_2(1−*x*)_Se_2*x*_ in the HER process. To understand the reason for the improved reactivity of MoS_2(1−*x*)_Se_2*x*_/NiSe_2_ hybrid catalysts, we examined the intermediate structures (Fig. 4b). When the MoS_2(1−*x*)_Se_2*x*_ particles are placed on top of the NiSe_2_ substrate, they relax to form a chemically bonded hybrid on the (100) and (110) surfaces of NiSe_2_, while remaining unbonded from the (111) surface of NiSe_2_. Thus, DFT calculations corroborate that the MoS_2(1−*x*)_Se_2*x*_/NiSe_2_ hybrid is a promising electrocatalyst (Fig. 4).

## Discussion

In general, the as-prepared hybrid catalysts possess the merits of all the MoS_2_ catalysts that have ever been reported on improving the relevant catalytic performance^18^,^19^. Namely, the outstanding HER activity as well as good stability for ternary MoS_2(1−*x*)_Se_2*x*_ particles/porous NiSe_2_ foam can be attributed to the synergistic effects from the dense catalytic edge sites at the MoS_2(1−*x*)_Se_2*x*_ surface, good dispersion of the MoS_2(1−*x*)_Se_2*x*_ particles on NiSe_2_ foam, good electrical contact and chemical bonding between MoS_2(1−*x*)_Se_2*x*_ and NiSe_2_ catalysts, and 3D porous structures of HER-active NiSe_2_ foam: first, similar to MoS_2_, the catalytic property of MoS_2(1−*x*)_Se_2*x*_ is greatly related to the number of exposed edge sites^11^,^37^. Indeed, in our experiments, because of the porous structure and curved surface of as-grown NiSe_2_ foam, ternary MoS_2(1−*x*)_Se_2*x*_ layers tend to exhibit vertical orientation on the NiSe_2_ surface as demonstrated using high-resolution TEM, indicating that abundant active edge sites are exposed in these MoS_2(1−*x*)_Se_2*x*_ particles^27^. Second, the electrical conductivity of MoS_2(1−*x*)_Se_2*x*_ catalysts is another crucial factor to the electrocatalytic activity since the intrinsic conductivity is extremely low between two adjacent van der Waals bonded S–Mo–S layers^14^,^16^. In our case, the MoS_2(1−*x*)_Se_2*x*_ layers are vertically oriented, which enhances the electron transfer from the electrode to the MoS_2(1−*x*)_Se_2*x*_ layers. Furthermore, the underlying NiSe_2_ foam is metallic and is composed of lots of 3D porous structures that ensure rapid electron transport from the less-conducting MoS_2(1−*x*)_Se_2*x*_ to the electrodes, and easy diffusion of the electrolyte into the active sites^18^. Finally, the Gibbs free energy for hydrogen adsorption on MoS_2(1−*x*)_Se_2*x*_ edges plays significant roles in this HER process, which is much lower compared with MoS_2_, leading to higher coverage of hydrogen adsorption at the active sites. Thus, by using this strategy, we can simultaneously engineer the catalysts with high surface area, 3D porous structures, good electrical conductivity and a large number of exposed active edge sites.

In summary, we propose and validate a simple and efficient strategy to synthesize a robust and stable self-standing hydrogen-evolving catalyst by simply growing ternary MoS_2(1−*x*)_Se_2*x*_ particles on 3D porous and metallic NiSe_2_ foam. According to our experimental results and quantum mechanics DFT calculation, these MoS_2(1−*x*)_Se_2*x*_/NiSe_2_ hybrid catalysts exhibit an outstanding catalytic performance superior to that of the widely reported LTMD catalysts (especially MoS_2_, WS_2_ and so on) and first-row transition metal pyrites (CoSe_2_, CoS_2_ and so on). Our catalysts are very effective in catalysing hydrogen production by integrating metal dichalcogenides and pyrites into 3D hybrid architectures that possess high surface area, porous structures, good electrical conductivity and abundant active edge sites, making it promising to realize large-scale water splitting.

## Methods

### Material synthesis

3D porous NiSe_2_ foam was directly synthesized by thermal selenization of commercial Ni foam in a tube furnace. Then, the as-prepared NiSe_2_ foam was immersed in (NH_4_)_2_MoS_4_ solution in dimethylformamide (DMF) solvent (5 wt% (NH_4_)_2_MoS_4_ in DMF) and dried on the hot plate, followed by thermolysis or second selenization at 500 °C in the tube furnace. The details are shown in the Supplementary Information.

### Electrochemical measurements

The electrochemical measurements were conducted in a three-electrode setup with an electrochemical station (Gamry, Reference 600). The polarization curves were collected by linear sweep voltammetry with a scan rate of 0.5 mV s^−1^ in 82 ml of 0.5 M H_2_SO_4_, so as to suppress the capacitive current due to the high surface area and high porosity of the porous samples^38^. A saturated calomel electrode was used as the reference electrode, a Pt wire (CH Instruments Inc.) as the counter electrode and as-prepared hybrid catalysts as the self-supported working electrodes. During the electrochemical measurements, high-purity N_2_ gas was continually bubbled throughout the whole electrochemical measurement. Potentials versus RHE can be calculated compared with saturated calomel electrode by adding a value of 0.263 V after calibration. The electrochemical stability of the catalyst was evaluated by continuously cycling the catalyst for 1,000 times at a scan rate of 50 mV s^−1^. Chronoamperometry was performed under a given potential for the MoS_2(1−*x*)_Se_2*x*_/NiSe_2_ hybrid electrode. The electrochemical impedance spectroscopy test was carried out in the same device configuration at a potential of −0.15 V versus RHE with the frequency ranging from 10 mHz to 1 MHz with a 10 mV AC dither. All the potentials used here were referred to RHE.

### Data availability

The data that support the findings of this study are available from the corresponding author upon request.

## Additional information

**How to cite this article:** Zhou, H. *et al.* Efficient hydrogen evolution by ternary molybdenum sulfoselenide particles on self-standing porous nickel diselenide foam. *Nat. Commun.* 7:12765 doi: 10.1038/ncomms12765 (2016).

## Supplementary Material

Supplementary Information Supplementary Figures 1-7, Supplementary Tables 1-3, Supplementary Notes 1-6 and Supplementary References.

## References

[b1] Bockris J. O. M. The origin of ideas on a hydrogen economy and its solution to the decay of the environment. Int. J. Hydrogen Energy 27, 731–740 (2002).

[b2] Yang Y. *et al.* A hybrid energy cell for self-powered water splitting. Energy Environ. Sci. 6, 2429–2434 (2013).

[b3] Gratzel M. Photoelectrochemical cells. Nature 414, 338–344 (2001). 11713540 10.1038/35104607

[b4] Cook T. R. *et al.* Solar energy supply and storage for the legacy and nonlegacy worlds. Chem. Rev. 110, 6474–6502 (2010). 21062098 10.1021/cr100246c

[b5] Liu G. *et al.* Heteroatom-modulated switching of photocatalytic hydrogen and oxygen evolution preferences of anatase TiO_2_ microspheres. Adv. Funct. Mater. 22, 3233–3238 (2012).

[b6] Trasatti S. Electrocatalysisof hydrogen evolution: progress in cathode activation. Adv. Electrochem. Sci. Eng. 2, 1–85 (1992).

[b7] Lu Y. C., Gasteiger H. A. & Shao-Horn Y. Catalytic activity trends of oxygen reduction reaction for nonaqueous Li-air batteries. J. Am. Chem. Soc. 133, 19048–19051 (2011). 22044022 10.1021/ja208608s

[b8] Tang W. *et al.* Self-powered water splitting using flowing kinetic energy. Adv. Mater. 27, 272–276 (2015). 25413298 10.1002/adma.201404071

[b9] Faber M. S. & Jin S. Earth-abundant inorganic electrocatalysts and their nanostructures for energy conversion applications. Energy Environ. Sci. 7, 3519–3542 (2014).

[b10] Morales-Guio C. G., Stern L. A. & Hu X. L. Nanostructured hydrotreating catalysts for electrochemical hydrogen evolution. Chem. Soc. Rev. 43, 6555–6569 (2014). 24626338 10.1039/c3cs60468c

[b11] Hinnemann B. *et al.* Biomimetic hydrogen evolution: MoS_2_ nanoparticles as catalyst for hydrogen evolution. J. Am. Chem. Soc. 127, 5308–5309 (2005). 15826154 10.1021/ja0504690

[b12] Gao M. R., Xu Y. F., Jiang J. & Yu S. H. Nanostructured metal chalcogenides: synthesis, modification, and applications in energy conversion and storage devices. Chem. Soc. Rev. 42, 2986–3017 (2013). 23296312 10.1039/c2cs35310e

[b13] Karunadasa H. I. *et al.* A molecular MoS_2_ edge site mimic for catalytic hydrogen generation. Science 335, 698–702 (2012). 22323816 10.1126/science.1215868

[b14] Lukowski M. A. *et al.* Enhanced hydrogen evolution catalysis from chemically exfoliated metallic MoS_2_ nanosheets. J. Am. Chem. Soc. 135, 10274–10277 (2013). 23790049 10.1021/ja404523s

[b15] Lukowski M. A. *et al.* Highly active hydrogen evolution catalysis from metallic WS_2_ nanosheets. Energy Environ. Sci. 7, 2608–2613 (2014).

[b16] Xie J. F. *et al.* Controllable disorder engineering in oxygen- incorporated MoS_2_ ultrathin nanosheets for efficient hydrogen evolution. J. Am. Chem. Soc. 135, 17881–17888 (2013). 24191645 10.1021/ja408329q

[b17] Li Y. *et al.* MoS_2_ nanoparticles grown on graphene: an advanced catalyst for the hydrogen evolution reaction. J. Am. Chem. Soc. 133, 7296–7299 (2011). 21510646 10.1021/ja201269b

[b18] Li D. J. *et al.* Molybdenum sulfide/N-doped CNT forest hybrid catalysts for high- performance hydrogen evolution reaction. Nano Lett. 14, 1228–1233 (2014). 24502837 10.1021/nl404108a

[b19] Kibsgaard J., Chen Z. B., Reinecke B. N. & Jaramillo T. F. Engineering the surface structure of MoS_2_ to preferentially expose active edge sites for electrocatalysis. Nat. Mater. 11, 963–969 (2012). 23042413 10.1038/nmat3439

[b20] Xu K. *et al.* Component-controllable WS_2(1–x)_Se_2x_ nanotubes for efficient hydrogen evolution reaction. ACS Nano 8, 8468–8476 (2014). 25110810 10.1021/nn503027k

[b21] Gao M. R. *et al.* An efficient molybdenum disulfide/cobalt diselenide hybrid catalyst for electrochemical hydrogen generation. Nat. Commun. 6, 5982 (2015). 25585911 10.1038/ncomms6982 PMC4309426

[b22] Wang X. G., Kolen'ko Y. V., Bao X. Q., Kovnir K. & Liu L. F. One-step synthesis of self-supported nickel phosphide nanosheet array cathodes for efficient electrocatalytic hydrogen generation. Angew. Chem. Int. Ed. 54, 8188–8192 (2015). 10.1002/anie.201502577 26032688

[b23] Cabán-Acevedo M. *et al.* Efficient hydrogen evolution catalysis using ternary pyrite-type cobalt phosphosulphide. Nat. Mater. 14, 1245–1251 (2015). 26366849 10.1038/nmat4410

[b24] Kong D. S., Wang H. T., Lu Z. Y. & Cui Y. CoSe_2_ nanoparticles grown on carbon fiber paper: an efficient and stable electrocatalyst for hydrogen evolution reaction. J. Am. Chem. Soc. 136, 4897–4900 (2014). 24628572 10.1021/ja501497n

[b25] Chen Z. P. *et al.* Three-dimensional flexible and conductive interconnected graphene networks grown by chemical vapour deposition. Nat. Mater. 10, 424–428 (2011). 21478883 10.1038/nmat3001

[b26] Zhou H. Q. *et al.* One-step synthesis of self-supported porous NiSe_2_/Ni hybrid foam: an efficient 3D electrode for hydrogen evolution reaction. Nano Energy 20, 29–36 (2016).

[b27] Wang H. T. *et al.* MoSe_2_ and WSe_2_ nanofilms with vertically aligned molecular layers on curved and rough surfaces. Nano Lett. 13, 3426–3433 (2013). 23799638 10.1021/nl401944f

[b28] Gong Y. J. *et al.* Band gap engineering and layer-by-layer mapping of selenium-doped molybdenumd disulfide. Nano Lett. 14, 442–449 (2014). 24368045 10.1021/nl4032296

[b29] Li H. L. *et al.* Growth of alloy MoS_2x_Se_2(1–x)_ nanosheets with fully tunable chemical compositions and optical properties. J. Am. Chem. Soc. 136, 3756–3759 (2014). 24564365 10.1021/ja500069b

[b30] Wang F. M. *et al.* Enhanced electrochemical H_2_ evolution by few-layered metallic WS_2(1-x)_Se_2x_ nanoribbons. Adv. Funct. Mater. 25, 6077–6083 (2015).

[b31] Peng S. J. *et al.* Cobalt sulfide nanosheet/ graphene/carbon nanotube nanocomposites as flexible electrodes for hydrogen evolution. Angew. Chem. Int. Ed. 126, 12802–12807 (2014). 10.1002/anie.201408876 25297454

[b32] Benck J. D., Chen Z. B., Kuritzky L. Y., Forman A. J. & Jaramillo T. F. Amorphous molybdenum sulfide catalysts for electrochemical hydrogen production: insights into the origin of their catalytic activity. ACS Catal. 2, 1916–1923 (2012).

[b33] Tsai C., Chan K., Nørskov J. K. & Abild-Pedersen F. Theoretical insights into the hydrogen evolution activity of layered transition metal dichalcogenides. Surface Sci. 640, 133–140 (2015).

[b34] Huang Y. F., Nielsen R. J., Goddard III W. A. & Soriaga M. P. The reaction mechanism with free energy barriers for electrochemical dihydrogen evolution on MoS_2_. J. Am. Chem. Soc. 137, 6692–6698 (2015). 25941943 10.1021/jacs.5b03329

[b35] Gong Q. F. *et al.* Ultrathin MoS_2(1–x)_Se_2x_ alloy nanoflakes for electrocatalytic hydrogen evolution reaction. ACS Catal. 5, 2213–2219 (2015).

[b36] Kiran V., Mukherjee D., Jenjeti R. N. & Sampath S. Active guests in the MoS_2_/MoSe_2_ host lattice: efficient hydrogen evolution using few-layer alloys of MoS_2(1-x)_Se_2x_. Nanoscale 6, 12856–12863 (2014). 25230335 10.1039/c4nr03716b

[b37] Jaramillo T. F. *et al.* Identification of active edge sites for electrochemical H_2_ evolution from MoS_2_ nanocatalysts. Science 317, 100–102 (2007). 17615351 10.1126/science.1141483

[b38] Lu Q. *et al.* Highly porous non-precious bimetallic electrocatalysts for efficient hydrogen evolution. Nat. Commun. 6, 6567 (2015). 25910892 10.1038/ncomms7567 PMC4382682

