# Supplementary material for: Efficient hydrogen evolution by ternary molybdenum sulfoselenide particles on self-standing porous nickel diselenide foam
Source: Nat Commun. 2016 Sep 16;7:12765. doi: 10.1038/ncomms12765 (PMC5028416; doi:10.1038/ncomms12765)
Supplement: Supplementary Information — Supplementary Figures 1-7, Supplementary Tables 1-3, Supplementary Notes 1-6 and Supplementary References. [file ncomms12765-s1.pdf]

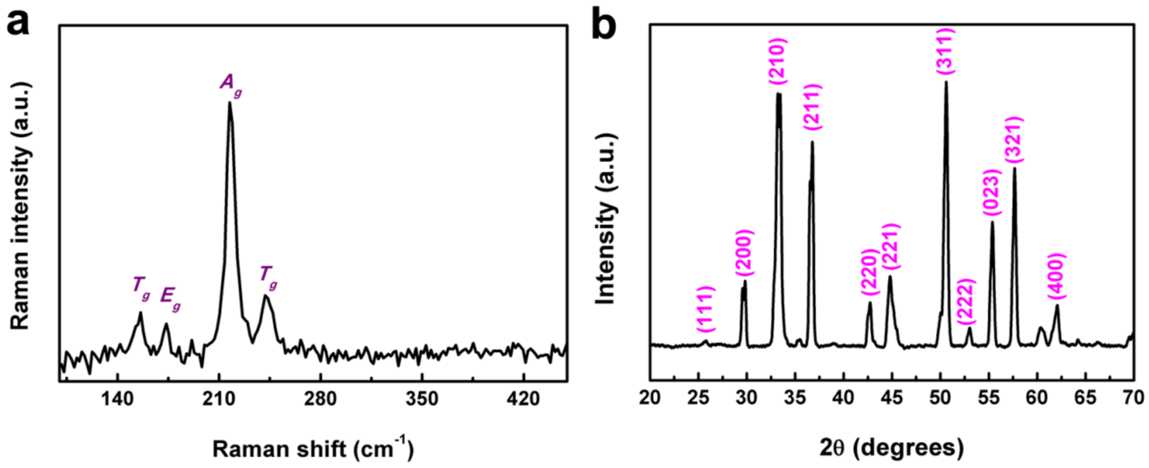

Supplementary Figure 1. (a) A typical Raman spectrum to confirm the selenization of commercial Ni foam into $NiSe_2$ foam. (b) A typical X-ray diffraction pattern of the as-prepared $NiSe_2$ foam.

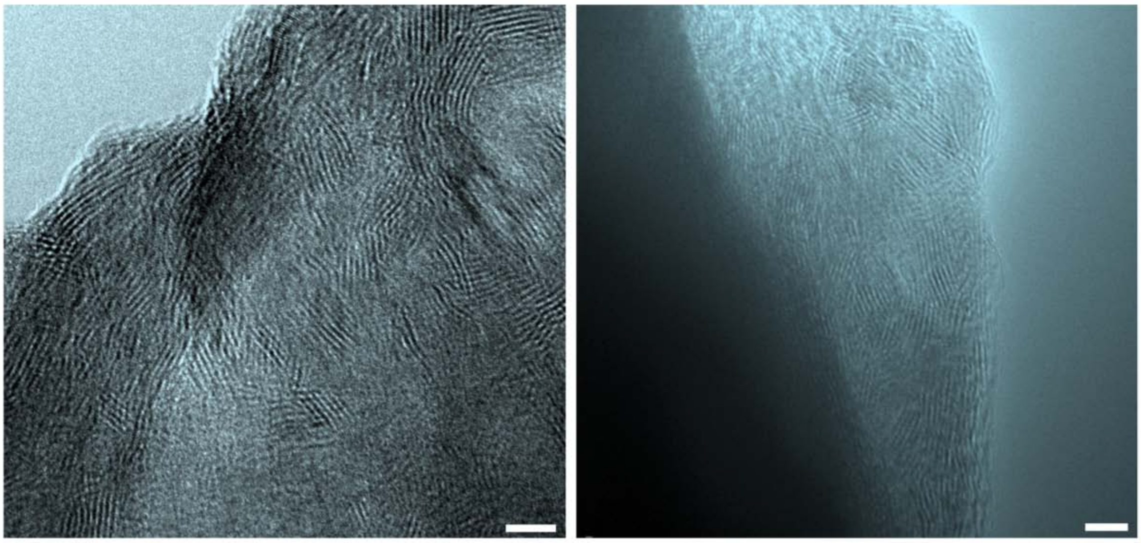

Supplementary Figure 2. TEM images showing the vertical layer orientation of $MoS_{2(1-x)}Se_{2x}$ particles grown on porous $NiSe_2$ foam. Scale bar: 5 nm.

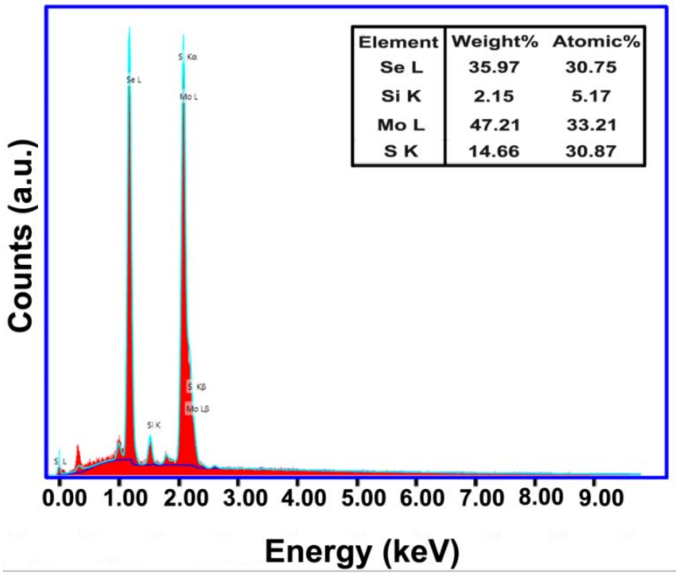

Supplementary Figure 3. EDS analysis on the chemical composition of as-prepared $MoS_{2(1-x)}Se_{2x}$ particles on Si.

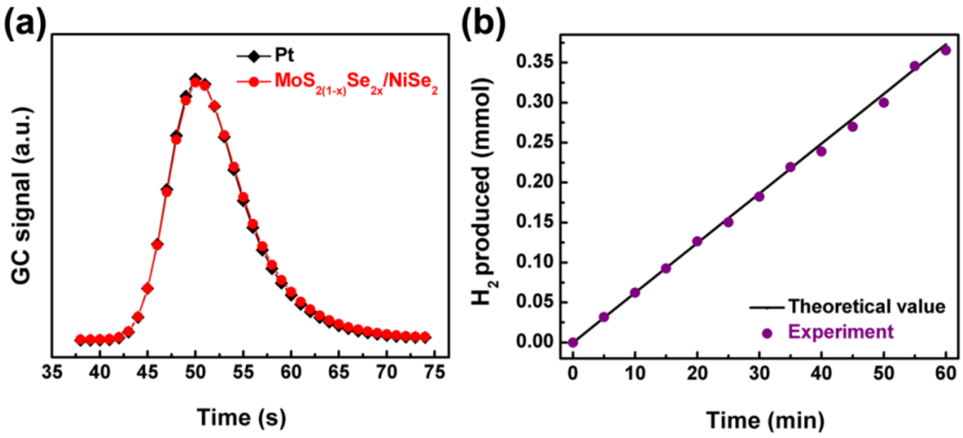

Supplementary Figure 4. Faraday efficiency measurement. (a) GC signals for the $MoS_{2(1-x)}Se_{2x}/NiSe_2$ hybrid electrode and Pt reference electrode after 5 min reaction. (b) The amount of H_2 theoretically calculated (solid) and experimentally measured (sphere) versus time for $MoS_{2(1-x)}Se_{2x}/NiSe_2$ hybrid catalyst at a constant current density of - 40 mA cm^{-2}. It is shown that there is a good correlation between the calculated and experimental amounts of H_2 gas, indicating near 100% Faraday efficiency.

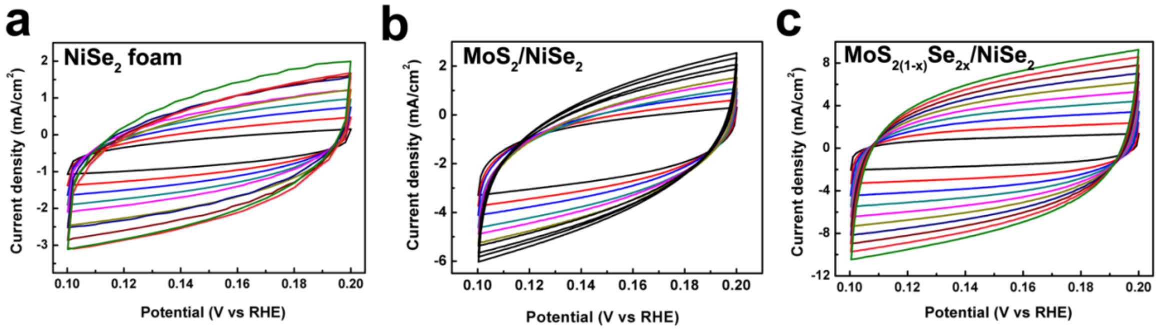

Supplementary Figure 5. Electrochemical cyclic voltammetry curves of as-grown catalysts at different potential scanning rates. (a) Original $NiSe_2$ foam grown at 600 ^{o}C with scan rates from 20 mV s^{-1} to 200 mV s^{-1} with an interval point of 20 mV s^{-1}. (b) $MoS_2/NiSe_2$ hybrid with scan rates from 15 mV s^{-1} to 60 mV s^{-1} with a 5 mV s^{-1} interval. (c) $MoS_{2(1-x)}Se_{2x}/NiSe_2$ foam with porous $NiSe_2$ foam grown at 600 ^{o}C and $MoS_{2(1-x)}Se_{2x}$ grown at 500 ^{o}C. For $MoS_{2(1-x)}Se_{2x}/NiSe_2$ hybrid, the scan rates range from 2 mV s^{-1} to 20 mV s^{-1} with an interval point of 2 mV s^{-1}.

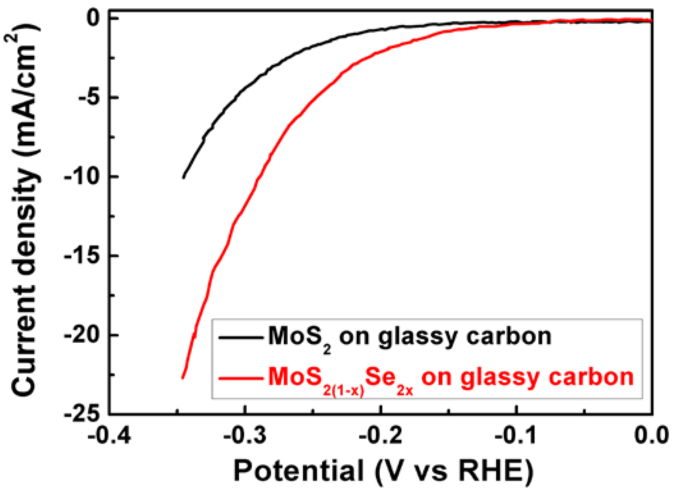

Supplementary Figure 6. The polarization curves recorded on $MoS_{2(1-x)}Se_{2x}$ or MoS_2 particles loaded on glassy carbon electrodes. The catalyst loading of $MoS_{2(1-x)}Se_{2x}$ or MoS_2 particles is 0.285 mg cm^{-2}.

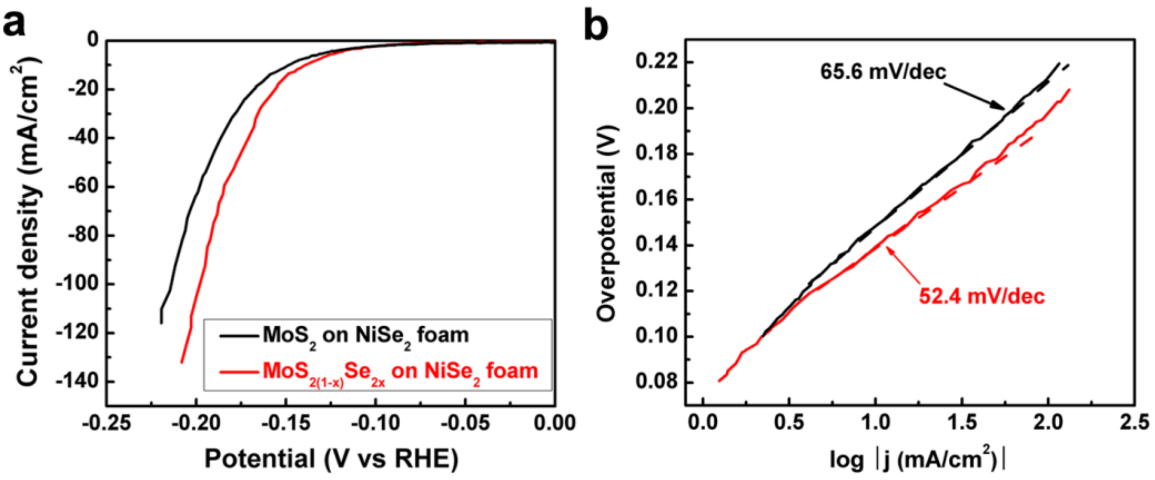

Supplementary Figure 7. The comparison on the catalytic performance between $MoS_{2(1-x)}Se_{2x}$ and MoS_2 particles loaded on porous $NiSe_2$ foam. Porous $NiSe_2$ foams were grown at the same conditions with the growth temperature at 600 ^oC. The loading of $MoS_{2(1-x)}Se_{2x}$ or MoS_2 catalyst is 0.6 mg cm^{-2}.

Supplementary Table 1. The detailed parameters for the catalytic HER performance of different catalysts examined in Figure 3. Here j_0 represents the exchange current density, while, η_{10}, η_{20} and η_{100} are corresponding to the potentials vs RHE at 10 mA cm^{-2}, 20 mA cm^{-2} and 100 mA cm^{-2} current densities, respectively.

Catalyst	Tafel slope	j_0	η_{10}	η_{20}	η_{100}
$MoS_{2(1-x)}Se_{2x}/NiSe_2$	42.1 mV dec^{-1}	299.4 µA cm^{-2}	69 mV	80 mV	112 mV
$MoS_2/NiSe_2$	58.5 mV dec^{-1}	104.9 µA cm^{-2}	118 mV	136 mV	189 mV
Pure $NiSe_2$	46.4 mV dec^{-1}	11.4 µA cm^{-2}	153 mV	166 mV	198 mV
Pt wire	30.0 mV dec^{-1}	1078 µA cm^{-2}	32 mV	43 mV	71 mV

Supplementary Table 2. The comparison on the catalytic performance of our catalysts with other low-cost HER electrocatalysts available from literatures. All the catalysts referred here are tested in 0.5 M H_2SO_4 electrolytes. Here j_0 is the exchange current density, while η_{10}, η_{20} and η_{100} correspond to the potentials at 10 $mAcm^{-2}$, 20 $mAcm^{-2}$ and 100 $mAcm^{-2}$ current densities, respectively.

**Catalyst**	**Tafel slope**	η_{10}	η_{20}	η_{100}	j_0	**Reference**
$MoS_{2(1-x)}Se_{2x}/NiSe_2$	42 mV dec^{-1}	69 mV	80 mV	112 mV	299 μA cm^{-2}	This work
MoS_x/N-CNT	40 mV dec^{-1}	110 mV	128 mV	225 mV	33.1 μA cm^{-2}	1
Double-gyroid MoS_2	50 mV dec^{-1}	240 mV			0.7 μA cm^{-2}	2
Li-MoS_2	62 mV dec^{-1}	118 mV	135 mV	175 mV	63 μA cm^{-2}	3
CoS_2/RGO-CNT	51 mV dec^{-1}	142 mV	153 mV	178 mV	62.6 μA cm^{-2}	4
FeS_2 nanosheets	46 mV dec^{-1}	108 mV	120 mV	170 mV	5.5 μA cm^{-2}	5
$CoSe_2$/carbon fiber	42 mV dec^{-1}	139 mV	155 mV	184 mV	6 μA cm^{-2}	6
MoS_2 nanosheets	43 mV dec^{-1}	187 mV	200 mV	240 mV	0.5 μA cm^{-2}	7
WS_2 nanosheets	55 mV dec^{-1}	240 mV	280 mV		20 μA cm^{-2}	8
$WS_{1.56}Se_{0.44}$ nanoribbons	68 mV dec^{-1}	176 mV				9
Ni_5P_4-Ni_2P nanosheets	79 mV dec^{-1}	120 mV	140 mV	200 mV	116 μA cm^{-2}	10
MoP particles	54 mV dec^{-1}	140 mV	160 mV	350 mV	34 μA cm^{-2}	11
Ni_2P nanoparticles	46 mV dec^{-1}	105 mV	130 mV	180 mV	33 μA cm^{-2}	12
CoP nanowire array/CC	51 mV dec^{-1}	67 mV	100 mV	204 mV	288 μA cm^{-2}	13
MoC_x nano-octahedrons	53 mV dec^{-1}	142 mV	163 mV	240 mV	23 μA cm^{-2}	14
Metallic FeNiS nanosheet	40 mV dec^{-1}	105 mV	140 mV	180 mV	20 μA cm^{-2}	15

Supplementary Table 3. The comparison on the TOF values of our catalysts with other low-cost HER electrocatalysts available from literatures.

**Catalyst**	***Potential***	***TOF***	**Active site density**	**Source**
$MoS_{2(1-x)}Se_{2x}/NiSe_2$ hybrid	100 mV	$0.010\text{-}0.030\ s^{-1}$	$(0.585\text{-}1.755) \times 10^{19}\ cm^{-2}$	This work
	125 mV	$0.030\text{-}0.091\ s^{-1}$		
	150 mV	$0.073\text{-}0.219\ s^{-1}$		
Ni_2P nanoparticles	100 mV	$0.015\ s^{-1}$	$8.17 \times 10^{17}\ cm^{-2}$	12
	200 mV	$0.5\ s^{-1}$		
CoP nanoparticles	100 mV	$0.046\ s^{-1}$	$1.65 \times 10^{18}\ cm^{-2}$	16
MoS_2 nanosheets	300 mV	$0.725\ s^{-1}$		17
Li-MoS_2	200 mV	$0.1\ s^{-1}$	$6.4 \times 10^{18}\ cm^{-2}$	3
MoS_x/N-CNT	200 mV	$3.5\ s^{-1}$	$\sim 9.8 \times 10^{16}\ cm^{-2}$	1
Double-gyroid MoS_2	150 mV	$0.08\ s^{-1}$	$\sim 2.9 \times 10^{17}\ cm^{-2}$	2
MoS_2/RGO	150 mV	$0.035\ s^{-1}$		18
Mo-W-P/carbon cloth	100 mV	$0.02\ s^{-1}$	$\sim 2.98 \times 10^{18}\ cm^{-2}$	19
	150 mV	$0.15\ s^{-1}$		
MoP｜S film	150 mV	$0.7\ s^{-1}$	$5.15 \times 10^{17}\ cm^{-2}$	20
MoP film	150 mV	$0.18\ s^{-1}$	$6.08 \times 10^{17}\ cm^{-2}$	20
MoC_x nano-octahedrons	200 mV	$0.057\ s^{-1}$		14
FeS_2 nanosheets	170 mV	$0.31\ s^{-1}$	$2.02 \times 10^{16}\ cm^{-2}$	5

Supplementary Note 1: Synthesis of porous $NiSe_2$ foam from commercial Ni foam.

The synthesis of porous $NiSe_2$ foam was performed via direct selenization in a tube furnace. The commercial Ni foam was cut into pieces with an area of 1.0 cm^2. Selenium powder (99.5%, Alfa Aesar) was used to supply Se vapor at the upstream of the furnace, which was transferred to the center region by Ar gas and reacted with Ni foam. System purging was performed before heating by introducing high-flow Ar gas (99.999%, ultrahigh purity). The furnace was programmed and heated to 600 ^{o}C in a short time and kept unchanged for 1h. Finally, the tube furnace was automatically turned off and cooled down with the protection of Ar gas.

Supplementary Note 2: Growth of $MoS_{2(1-x)}Se_{2x}$ or MoS_2 particles on porous $NiSe_2$ foam.

The precursor ammonium tetrathiomolybdate ($(NH_4)_2MoS_4$) was purchased from Sigma Aldrich. The as-prepared $NiSe_2$ foam was immersed in $(NH_4)_2MoS_4$ solution in dimethylformamide (DMF) solvent (5 wt% $(NH_4)_2MoS_4$ in DMF). The samples were then baked on a hot plate at 100 ° C for 30 min. To grow pure MoS_2 or ternary $MoS_{2(1-x)}Se_{2x}$ particles, the samples were placed at the center of a tube furnace for thermolysis or a second selenization at 500 ^{o}C in pure Ar atmosphere.

Supplementary Note 3: Material characterization.

The micro-Raman spectroscopy (Renishaw inVia Raman Spectroscope) experiments were performed to characterize different $NiSe_2$ foam-based samples with a 514 nm laser. XPS spectra were collected using a PHI Quantera SXM Scanning X-ray Microprobe under 5×10^{-9} Torr, and the data were analyzed by a MultiPak software. The morphologies of the catalysts were investigated by scanning electron microscope (SEM) (LEO 1525). The detailed structure of the as-grown material was imaged by a

high-resolution transmission electron microscope (TEM, JEOL 2010F). The TEM samples were strips cut from $MoS_{2(1-x)}Se_{2x}/NiSe_2$ foam without any grinding to preserve the intrinsic morphology of $MoS_{2(1-x)}Se_{2x}$ particles with vertically aligned layers.

Supplementary Note 4: Faraday efficiency measurements.

Gas chromatography (GC) technique was used to quantify the Faraday efficiency of the $MoS_{2(1-x)}Se_{2x}/NiSe_2$ hybrid catalyst. Hydrogen (H_2) was first generated in a sealed electrochemical cell (Gamry Reference 600) under a constant cathodic current density of - 40 mA cm^{-2}. For each measurement, 0.25 mL gas sample was taken from the sealed cell and injected into the GC instrument (GOW-MAC 350 TCD) using a glass syringe (Hamilton Gastight 1002). The GC signal was read by a Keithley 2400 Source Meter connected with PC, and the data was recorded in a LabVIEW program. The generation of H_2 gas was further proven by comparing the GC signals of H_2 between the hybrid catalyst and a Pt wire. The amount of H_2 is almost the same between these two catalysts during the same reaction time period (Supplementary Figure 4a).

Supplementary Note 5: Calculation of turn over frequency (TOF).

For rough estimation of the active surface site density and per-site turn over frequency (TOF) in the $MoS_{2(1-x)}Se_{2x}/NiSe_2$ hybrid catalyst, we suppose that the contribution of the $MoS_{2(1-x)}Se_{2x}$ particles plays a dominant role. This is reasonable since the surface of $NiSe_2$ foam is nearly fully covered by the $MoS_{2(1-x)}Se_{2x}$ particles, and the catalytic performance of ternary $MoS_{2(1-x)}Se_{2x}/NiSe_2$ hybrid is far better than that of pure $NiSe_2$ foam. According to this approach adopted by Jaramillo et al[20,21], we carried out a similar calculation method by considering the relative roughness factor of the catalyst, the geometry of a

$MoS_{2(1-x)}Se_{2x}$ surface, and the hydrogen evolution current density. As shown in Fig. 3e, we have determined the specific capacitance to be 319 mF cm^{-2}, which can be directly used to estimate the relevant electrochemical active surface area (ECSA) by using the specific capacitance value for a flat electrode with real surface area 1 cm^2. We assume 60 μF cm^{-2} for a flat electrode provided in Jaramillo et al[2,21] and Kim et al[1] for calculation here, and use 20 and 60 μF cm^{-2} for evaluating a lower and upper limit of the TOFs[20] (Supplementary Table 3). Thus, the number of electrochemically effective surface sites on the $MoS_{2(1-x)}Se_{2x}$ catalyst was calculated as the following:

$$\frac{\#\, Surface\ sites\ (catalyst)}{cm^2\ geometric\ area} = \frac{\#\, Surface\ sites\ (flat\ standard)}{cm^2\ geometric\ area} \times Roughness\ factor \quad (1)$$

Compared to the flat standard electrode (60 μF cm^{-2}), the relative roughness factor of the investigated catalyst is determined to be ~ 5316 based on the electrochemically double-layer capacitance measurement. As a result, the number of surface active sites for the $MoS_{2(1-x)}Se_{2x}/NiSe_2$ hybrid catalyst is estimated to be 5.85×10^{18} surface sites/cm^2 from the above formula, indicating a large number of active sites introduced by our special experimental design.

To further get insights into the per-site TOF, the following formula is utilized:

$$TOF\ per\ site = \frac{\#\, Total\ Hydrogen\ Turn\ Overs/cm^2 geometric\ area}{\#\, Surface\ Sites\ (Catalyst)/cm^2\ geometric\ area} \quad (2)$$

The total number of hydrogen turn overs is related to the current density, and is calculated based on the following conversion:

$$\#_{H_2} = \left(j\,\frac{mA}{cm^2}\right)\left(\frac{1C\ s^{-1}}{1000\ mA}\right)\left(\frac{1\ mol\ e^-}{96485.3\ C}\right)\left(\frac{1\ mol\ H_2}{2\ mol\ e^-}\right)\left(\frac{6.022\ \times 10^{23}\ H_2\ molecules}{1\ mol\ H_2}\right) = 3.12\ \times 10^{15}\ \frac{H_2/s}{cm^2}\ per\ \frac{mA}{cm^2}\ (3)$$

So the *TOF* per site for our investigated hybrid catalyst at different overpotentials vs. RHE and pH = 0 is calculated as follows:

At η = 100 mV, $$\left(3.12\ \times 10^{15}\ \frac{H_2/s}{cm^2}\Big/\frac{mA}{cm^2}\right)\left(57\ \frac{mA}{cm^2}\right)\left(\frac{1\ cm^2}{5.85\times 10^{18}\ surface\ \ sites}\right) = 0.030\ \frac{H_2/\ s}{surface\ \ site}\ (4)$$

At η = 125 mV, $$\left(3.12\ \times 10^{15}\ \frac{H_2/s}{cm^2}\Big/\frac{mA}{cm^2}\right)\left(170\ \frac{mA}{cm^2}\right)\left(\frac{1\ cm^2}{5.85\times 10^{18}\ surface\ \ sites}\right) = 0.091\ \frac{H_2/\ s}{surface\ \ site}\ (5)$$

At η = 150 mV, $$\left(3.12\ \times 10^{15}\ \frac{H_2/s}{cm^2}\Big/\frac{mA}{cm^2}\right)\left(411\ \frac{mA}{cm^2}\right)\left(\frac{1\ cm^2}{5.85\times 10^{18}\ surface\ \ sites}\right) = 0.219\frac{H_2/\ s}{surface\ \ site}\ (6)$$

While for the $MoS_2/NiSe_2$ hybrid catalyst, given that its double-layer capacitance is around 30.9 $mFcm^{-2}$, and the current densities are 4.9, 13.0, and 31.0 $mAcm^{-2}$ at η = 100, 125, and 150 mV, respectively, we can get the corresponding TOF values to be 0.027, 0.071, and 0.170 H_2/s per surface site. These results help us to conclude that the $MoS_{2(1-x)}Se_{2x}/NiSe_2$ hybrid catalyst shows a faster TOF value compared to that of the $MoS_2/NiSe_2$ hybrid catalyst. As summarized above in *Supplementary Table 3*, the $MoS_{2(1-x)}Se_{2x}/NiSe_2$ hybrid catalyst has a faster TOF than that of MoS_2 on 3D carbon fiber paper,[3] MoS_2 particles on graphene oxide,[18] and double-gyroid MoS_2.[2] Meanwhile, by considering the loading (4.5 mg cm^{-2}) of $MoS_{2(1-x)}Se_{2x}$ particles, we can also make a rough estimation of the TOFs, which are 0.014, 0.041 and 0.099 s^{-1} at the overpotentials of 100, 125 and 150 mV, respectively.[14,22] These TOFs are in the range of the above values calculated by the capacitance method (Supplementary Table 3).

Supplementary Note 6: Computational methods.

All structures considered are optimized in VASP[23-26] using the PBE+D3[27-29] flavor of DFT. A kinetic energy cutoff of 300 eV for the planewave basis is used. The projected augmented wave (PAW) method[30,31] for pseudopotentials is employed to represent the contributions from core electrons. Free energies are calculated using PBE+D3 with CANDLE[32] implicit solvation in jDFTx[33-38] with GBRV uspp pseudopotentials. A higher kinetic energy cutoff of 13 Hartree (354 eV) is used for the free energy calculation. In order to compare the free energy difference under SHE condition, free energies are calculated at a fixed potential of 0 V vs. SHE. The expression for the free energy is $G = F - n_eU + ZPE + H_{vib} - TS_{vib}$, where ZPE (zero point energy), H_{vib} and S_{vib} are calculated from the vibrational contributions of the adsorbed hydrogen atom, F is the energy of the solvated Kohn-Sham DFT electronic system, n_e is the net explicit electrons in the system, and U is the chemical potential of electrons relative to vacuum at SHE. Since the hybrid $MoS_{2x}Se_{2(1-x)}/NiSe_2$ systems are large, as the smallest dimension of the periodic cell is greater than 15 A, a Γ-centered 1x1x1 Monkhorst-Pack k-point grid is used for all DFT calculations.

Supplementary References

1. Li, D. J. *et al*. Molybdenum sulfide/N-doped CNT forest hybrid catalysts for high- performance hydrogen evolution reaction. *Nano Lett.* **14**, 1228-1233 (2014).
2. Kibsgaard, J., Chen, Z. B., Reinecke, B. N. & Jaramillo, T. F. Engineering the surface structure of MoS_2 to preferentially expose active edge sites for electrocatalysis. *Nat. Mater.* **11**, 963-969 (2012).
3. Wang, H. T. *et al*. Electrochemical tuning of MoS_2 nanoparticles on three-dimensional substrate for efficient hydrogen evolution. *ACS Nano* **8**, 4940-4947 (2014).
4. Peng, S. J. *et al*. Cobalt sulfide nanosheet/graphene/carbon nanotube nanocomposites as flexible electrodes for hydrogen evolution. *Angew. Chem. Int. Ed.* **126**, 12802 -12807 (2014).
5. Wang, D. Y. *et al.* Highly active and stable hybrid catalyst of cobalt-doped FeS_2 nanosheets-carbon nanotubes for hydrogen evolution reaction. *J. Am. Chem. Soc.* **137**, 1587-1592 (2015).
6. Kong, D. S., Wang, H. T., Lu, Z. Y. & Cui, Y. $CoSe_2$ nanoparticles grown on carbon fiber paper: An efficient and stable electrocatalyst for hydrogen evolution reaction. *J. Am. Chem. Soc.* **136**, 4897-4900 (2014).
7. Lukowski, M. A. *et al*. Enhanced hydrogen evolution catalysis from chemically exfoliated metallic MoS_2 nanosheets. *J. Am. Chem. Soc*. **135**, 10274-10277 (2013).
8. Voiry, D. et al. Enhanced catalytic activity in strained chemically exfoliated WS_2 nanosheets for hydrogen evolution. *Nat. Mater.* **12**, 850-855 (2013).
9. Wang, F. M. *et al*. Enhanced electrochemical H_2 evolution by few-layered metallic $WS_{2(1-x)}Se_{2x}$ nanoribbons. *Adv. Funct. Mater.* **25**, 6077-6083 (2015).

10. Wang, X. G., Kolen'ko, Y. V., Bao, X. Q., Kovnir, K. & Liu, L. F. One-step synthesis of self-supported nickel phosphide nanosheet array cathodes for efficient electrocatalytic hydrogen generation. *Angew. Chem. Int. Ed.* **54**, 8188-8192 (2015).

11. Xiao, P. *et al*. Molybdenum phosphide as an efficient electrocatalyst for the hydrogen evolution reaction. *Energy Environ. Sci.* **7**, 2624-2629 (2014).

12. Popczun, E. J. *et al*. Nanostructured nickel phosphide as an electrocatalyst for the hydrogen evolution reaction. *J. Am. Chem. Soc.* **135**, 9267-9270 (2013).

13. Tian, J. Q., Liu, Q., Asiri, A. M. & Sun, X. P. Self-supported nanoporous cobalt phosphide nanowire arrays: An efficient 3D hydrogen-evolving cathode over the wide range of pH 0-14. *J. Am. Chem. Soc.* **136**, 7587-7590 (2014).

14. Wu, H. B., Xia, B. Y., Yu, L., Yu, X. Y. & Lou, X. W. Porous molybdenum carbide nano-octahedrons synthesized via confined carburization in metal-organic frameworks for efficient hydrogen production. *Nat. Commun.* **6**, 6512 (2015).

15. Long, X. *et al*. Metallic iron-nickel sulfide ultrathin nanosheets as a highly active electrocatalyst for hydrogen evolution reaction in acidic media. *J. Am. Chem. Soc.* **137**, 11900-11903 (2015).

16. Popczun, E. J., Read, C. G., Roske, C. W., Lewis, N. S. & Schaak, R. E. Highly active electrocatalysis of the hydrogen evolution reaction by cobalt phosphide nanoparticles. *Angew. Chem. Int. Ed.* **53**, 5427-5430 (2014).

17. Xie, J. F. et al. Defect-rich MoS_2 ultrathin nanosheets with additional active edge sites for enhanced electrocatalytic hydrogen evolution. *Adv. Mater.* **25**, 5807-5813 (2013).

18. Li, Y. G. et al. MoS_2 nanoparticles grown on graphene: An advanced catalyst for the hydrogen evolution reaction. *J. Am. Chem. Soc.* **133**, 7296-7299 (2011).

19. Wang, X. D. *et al*. Novel porous molybdenum tungsten phosphide hybrid nanosheets on carbon cloth for efficient hydrogen evolution. *Energy Environ. Sci.* **9**, 1468-1475 (2016).

20. Kibsgaard, J. & Jaramillo, T. F. Molybdenum phosphosulfide: An active, acid-stable, earth-abundant catalyst for the hydrogen evolution reaction. *Angew. Chem. Int. Ed.* **53**, 14433-14437 (2014).

21. Benck, J. D., Chen, Z. B., Kuritzky, L. Y., Forman, A. J. & Jaramillo, T. F. Amorphous molybdenum sulfide catalysts for electrochemical hydrogen production: Insights into the origin of their catalytic activity. *ACS Catal.* **2**, 1916-1923 (2012).

22. Fei, H. L. et al. Atomic cobalt on nitrogen-doped graphene for hydrogen generation. *Nat. Commun.* **6**, 8668 (2015).

23. Kresse, G. & Hafner, J. b initio molecular dynamics for liquid metals. *Phys. Rev. B: Condens. Matter Mater. Phys.* **47**, 558(R) (1993).

24. Kresse, G. & Hafner, J. Ab initio molecular-dynamics simulation of the liquid-metal–amorphous-semiconductor transition in germanium. *Phys. Rev. B: Condens. Matter Mater. Phys.* **49**, 14251 (1994).

25. Kresse, G. & Furthmuller, J. Efficiency of ab-initio total energy calculations for metals and semiconductors using a plane-wave basis set. *Comput. Mater. Sci.* **6**, 15-50 (1996).

26. Kresse, G. & Furthmuller, J. Efficient iterative schemes for ab initio total-energy calculations using a plane-wave basis set. Phys. *Rev. B: Condens. Matter Mater. Phys.* **54**, 11169 (1996).

27. Perdew, J. P., Burke, K. & Ernzerhof, M. Generalized gradient approximation made simple. *Phys. Rev. Lett.* **77**, 3865 (1996).

28. Perdew, J. P., Burke, K. & Ernzerhof, M. K^+ emission in symmetric heavy ion reactions at subthreshold energies. *Phys. Rev. Lett.* **78**, 1396 (1997).

29. Grimme, S., Antony, J., Ehrlich, S. & Krieg, S. A consistent and accurate ab initio parametrization of density functional dispersion correction (DFT-D) for the 94 elements H-Pu. *J. Chem. Phys.* **132**, 154104 (2010).

30. Blochl, P. E. Projector augmented-wave method. *Phys. Rev. B: Condens. Matter Mater. Phys.* **50**, 17953 (1994).

31. Kresse, G. & Joubert, D. From ultrasoft pseudopotentials to the projector augmented-wave method. *Phys. Rev. B: Condensed Matter Mater. Phys.* **59**, 1758 (1999).

32. Sundararaman, R. & Goddard, W. A. The charge-asymmetric nonlocally determined local-electric (CANDLE) solvation model. *J. Chem. Phys.* **142**, 064107 (2015).

33. Sundararaman, R., Gunceler, D., Letchworth-Weaver, K., Schwarz, K. A. & Arias, T. A. *JDFTx*, available from http://jdftx.sourceforge.net (2012).

34. Ismail-Beigi, S. & Arias, T. A. New algebraic formulation of density functional calculation. *Comput. Phys. Commun.* **128**, 1-45 (2000).

35. Rozzi, C. A., Varsano, D., Marini, A., Gross, E. K. U. & Rubio, A. Exact Coulomb cutoff technique for supercell calculations. *Phys. Rev. B.* **73**, 205119 (2006).

36. Petrosyan, S. A., Rigos, A. A. & Arias, T. A. Joint density-functional theory: Ab Initio study of

Cr_2O_3 surface chemistry in solution. *J. Phys. Chem. B*. **109**, 15436-15444 (2005).

37. Arias, T. A., Payne, M.C. & Joannopoulos, J. D. Ab initio molecular dynamics: Analytically continued energy functionals and insights into iterative solutions. *Phys. Rev. Lett.* **69**, 1077 (1992).

38. Freysoldt, C., Boeck, S. & Neugebauer, *J.* Direct minimization technique for metals in density functional theory. *Phys. Rev. B*. **79**, 241103(R) (2009).
